# Estimation of swine movement network at farm level in the US from the Census of Agriculture data

**DOI:** 10.1038/s41598-019-42616-w

**Published:** 2019-04-17

**Authors:** Sifat A. Moon, Tanvir Ferdousi, Adrian Self, Caterina M. Scoglio

**Affiliations:** 1 Department of Electrical & Computer Engineering, Kansas State University, Manhattan, Kansas United States of America; 2 National Agricultural Biosecurity Center, Kansas State University, Manhattan, Kansas United States of America

**Keywords:** Probabilistic data networks, Information technology

## Abstract

Swine movement networks among farms/operations are an important source of information to understand and prevent the spread of diseases, nearly nonexistent in the United States. An understanding of the movement networks can help the policymakers in planning effective disease control measures. The objectives of this work are: (1) estimate swine movement probabilities at the county level from comprehensive anonymous inventory and sales data published by the United States Department of Agriculture - National Agriculture Statistics Service database, (2) develop a network based on those estimated probabilities, and (3) analyze that network using network science metrics. First, we use a probabilistic approach based on the maximum information entropy method to estimate the movement probabilities among different swine populations. Then, we create a swine movement network using the estimated probabilities for the counties of the central agricultural district of Iowa. The analysis of this network has found evidence of the small-world phenomenon. Our study suggests that the US swine industry may be vulnerable to infectious disease outbreaks because of the small-world structure of its movement network. Our system is easily adaptable to estimate movement networks for other sets of data, farm animal production systems, and geographic regions.

## Introduction

Livestock are often moved between facilities to reduce costs and improve productivity. There is an old adage, “Livestock follow the grain”. Even now this aphorism seems true, as shipping animals is less expensive than shipping grains, which are required for animals to attain their slaughter weights. The corn-belt region (Iowa, Missouri, Illinois, Indiana, and Ohio states) is the largest market for feeder pigs^1^ because they are the largest producers of two major sources of hog rations (corn and soybeans). Although movements in the livestock industry can reduce the cost of production, movements have a major role in the risk of pathogens spread. Movement of swine among farms is one of the major pathways for the spread of several diseases (e.g., Porcine reproductive and respiratory syndrome-PRRS, Porcine epidemic diarrhea-PED etc.) in the United States (US) swine industry^2,3^. Knowledge of livestock movement can be useful in the control of pathogen spread. In Europe, there are several well-established animal tracking systems. However, similar programs are yet to be mandated for the US. In the US, a comprehensive livestock tracking system has not been implemented because of a cultural preference for privacy and competition between producers^4^. The United State Department of Agriculture (USDA) collects movement information when livestock shipments cross state boundaries. There is no program that collects movement information at the county or farm level.

In the prior literature, several models have been developed to understand swine movement in different regions of the US^4–6^. However, all of them used confidential incomplete datasets, which are not publicly accessible, and also which are not inclusive of the whole US. Yadav *et al*.^5^ developed a model to understand classical swine fever outbreak-related outcomes in Indiana. They used data from USAHerds (US Animal Health Emergency Reporting and Diagnostic System), where import-export activities, location of import origin, receiving swine premises, shipment size and shipment date are listed. However, only 22% of the states participates in the USAHerds program. Another research group predicted movement networks of the swine industry for some counties of Minnesota using a machine learning approach^6^. They used confidential survey data from two counties to train their model. The objective of our research is to understand the swine movement network in the US from publicly available data. A network is a useful structure in the study of any spreading phenomena, where farm-level animal movement networks are used as a key component in the area of disease spreading^7,8^.

In this work, we estimate the swine movement probabilities between counties based on published inventory and sales data from the USDA Census of Agriculture. We develop a convex optimization problem with some linear constraints for the US swine industry. To solve this problem, we adapt the cattle movement model from Schumm *et al*.^9^ for the swine population. In particular, we maximize the entropy of the distributions of the objective function (Eq. 1). Maximum information entropy methods have been used in various research fields^10–12^. The maximum entropy principle states that the best way to approximate the unknown distribution that satisfies all the constraints will have the maximum entropy^13^.

We propose a novel algorithm to develop a farm level swine movement network using the estimated swine movement probabilities. In this network, nodes (or vertices) represent swine-farms and directed links (or edges or connections) represent directional swine movements between the farms. Network realizations from the interactions among the elements of different dynamic systems can be seen several times in the literature; for example, weighted network for worldwide air transportation^14^, network for collaboration among scientists^14^, network to understand complex intercellular interactions^15^, and network to represent interplay among different physiological systems^16–19^. To understand the generated swine movement network, we use network centrality measures. They have been used often in the literature to understand the livestock movement patterns^20–22^. The network centrality measures can assist in detection of the important farms, which can control the movement flows in the network. This information can be useful to plan effective mitigation strategies to reduce an epidemic size. In the literature, researchers have used targeted vaccination, or quarantine, or culling of important agents to control epidemics^23,24^. The network centrality measure also can help us to understand the movement pattern. From the analysis of the developed swine movement network, we find a trace of the small world phenomenon and the presence of hubs in the US swine movement network.

## Materials and Methods

First, we develop a convex optimization problem to estimate swine movement probabilities. Next, we propose an algorithm to develop a network based on those probabilities, where nodes or vertices are farms or operations and edges among them represent swine movement. Finally, we analyze the network using different network analysis metrics.

### Data

We have collected the hog inventory, sales, slaughter, and dead/lost pig data from the United States Department of Agriculture National Agricultural Statistics Service (USDA-NASS)^25^. The USDA-NASS conducts a census every five years, which compiles a uniform, comprehensive agricultural data set for each county of the entire US. We used the data from the 2012 Census of Agriculture, as the census of 2017 is not published fully at the time of this research. For each county, two sets of data are available: (1) inventory and (2) sales. In both types, pigs are grouped into seven classes based on operation/farm size. These groups are: size1 (1–24 pigs), size2 (25–49 pigs), size3 (50–99 pigs), size4 (100–199 pigs), size5 (200–499 pigs), size6 (500–999 pigs), and size7 (more than 1000 pigs). For each size group, data for the number of operations and the number of pigs are available. However, several data points are not published to maintain anonymity; we estimate those to develop the network model. The study time of this research is the year 2012. We have assumed that the inventory sizes are constant throughout the year because of the resolution limitation of the available data. Another set of missing data are the geographic farm locations; we use geographical county centroids to measure the distances among counties.

We estimate the swine movement probabilities among sub-populations for the State of Iowa, where a sub-population is denoted as the swine population in a size group in a county. Iowa has the largest swine inventory (31.43%) in the US^25^. In the list of America’s top 100 pig farming counties, 42 counties are from Iowa alone^26^. It is also the most vulnerable state for the introduction of classical swine fever and African swine fever viruses due to legal import of live swine^27^. Iowa has 99 counties in total, the number of swine sub-populations in our optimization problem is 99 × 7.

### Swine movement probability estimation

To estimate the pig movement probabilities in a week among different sub-populations, we use a convex optimization problem. This convex optimization problem consists of two steps: (1) estimation of the non-disclosed data points in the inventory and sales data and (2) estimation of movement probabilities among different sub-populations.

**Figure 1:**
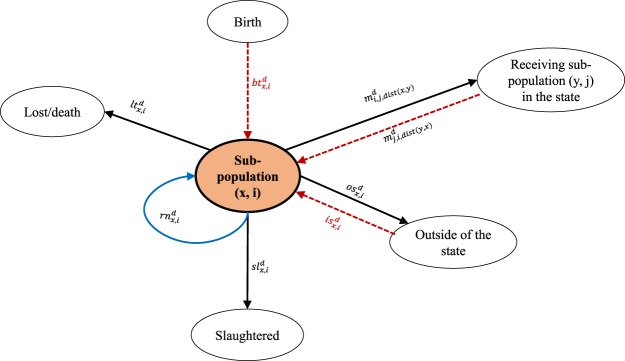
The movement flows of a sub-population (*x*, *i*). Solid black lines represent the outgoing flows from the sub-population, dotted red lines represent the incoming flows into the sub-population, and the blue solid line represents the possibility to stay or not moved. Solid lines (black and blue) form the distributions of the objective function. The probability of each movement are shown with the arrows.

To estimate non-disclosed points in the inventory data, we formulate an entropy function. By maximizing this function, we estimate the data points with minimum assumptions^28^. This process is detailed in Schumm *et al*.^9^. In step 2, we construct a convex optimization problem, which includes a series of linear constraints. The purpose of this problem is to maximize the entropy of the distributions of the objective function, the distributions of the objective function for a sub-population are presented in Fig. 1. The maximum entropy is a well-known method of statistical inference, which has been used in diverse research fields including ecology, thermodynamics, economics, forensics, language processing, astronomy, image processing etc.^12,29,30^. This method produces the least biased predictions while maintaining prior knowledge constraints.

In the convex optimization problem, there are *C* counties and each county has *I* size groups. A pig from a sub-population can be moved to a sub-population in the state, or moved outside of the state, or not moved at all, or slaughtered, or lost. Therefore, a pig in a sub-population has five movement options, which construct the distributions of the objective function. We define the objective function of this estimation problem as,

1 $$\begin{array}{rcl}max\{Entropy\} & = & max\{-\sum _{x\in C}\,\sum _{i\in I}\,\sum _{y\in C}\,\sum _{j\in I}\,{m}_{i,j,dist(x,y)}^{d}\ast log({m}_{i,j,dist(x,y)}^{d})\\  &  & -\,\sum _{x\in C}\,\sum _{i\in I}\,o{s}_{x,i}^{d}\ast log(o{s}_{x,i}^{d})-\sum _{x\in C}\,\sum _{i\in I}\,r{n}_{x,i}^{d}\ast log(r{n}_{x,i}^{d})\\  &  & -\,\sum _{x\in C}\,\sum _{i\in I}\,s{l}_{x,i}^{d}\ast log(s{l}_{x,i}^{d})-\sum _{x\in C}\,\sum _{i\in I}\,l{t}_{x,i}^{d}\ast log(l{t}_{x,i}^{d})\}\end{array}$$

The objective function of this problem is to maximize the *Entropy*. We estimate the movement probabilities ${m}_{i,j,dist(x,y)}^{d}$, which represents the movement probability from sub-population (*x*, *i*) to sub-population (*y*, *j*) in a week. A sub-population (*x*, *i*) is the swine population in the size group *i* in the county *x*. The index variable *x* and *i* are used for the originating sub-population, *x* = 1, 2, 3 … *C* and *i* = 1, 2, … *I*. Again, *y* and *j* are the index variable for the receiving sub-populations (*y*, *j*). The superscript *d* marks the decision parameters. The parameter $o{s}_{x,i}^{d}$ represents movement probability from sub-population (*x*, *i*) to outside of the state in a week, $r{n}_{x,i}^{d}$ is the probability to remain or not-moved in the sub-population (*x*, *i*) in a week, $s{l}_{x,i}^{d}$ is the probability of pigs being slaughtered for meat from sub-population (*x*, *i*) in a week, and $l{t}_{x,i}^{d}$ is the probability of pigs being dead or lost in sub-population (*x*, *i*) in a week. We divide the distance between counties into five classes: (1) distance ∈[0, 20), (2) distance ∈[20, 100), (3) distance ∈[100, 200), (4) distance ∈[200, 400), and (5) distance ∈[400, *D*_*max*_]. *D*_*max*_ is the maximum distance between two counties. *dist*(*x*, *y*) represents the distance class for the distance between county *x* and *y*. We divide the distances between all pairs of counties in that way to group them into discrete distance groups. This problem is subject to several linear constraints, which we construct from probability rules, sales data, swine population conservation etc.

As a pig can move (from the sub-population (*x*, *i*) to a sub-population in the state, or outside of the state, or slaughtered, or death) or it could stay in the sub-population, therefore the summation of these possibilities is equal to one. From the rule of the probability, we can get the following constraint for any sub-population (*x*, *i*),

2 $$\sum _{y\in C}\,\sum _{j\in I}\,{m}_{i,j,dist(x,y)}^{d}+o{s}_{x,i}^{d}+r{n}_{x,i}^{d}+s{l}_{x,i}^{d}+l{t}_{x,i}^{d}=1\,\,\forall (x,i)$$

The probabilities in Eq. 2 are considered in the objective function.

There are three types of sales in the system, (1) sales for the movement from sub-population (*x*, *i*) to the all sub-populations in the state, (2) sales for the movement to the outside of the state, and (3) sales for slaughter. Constraint for the sales or movement from any county *x* is,

3 $$\begin{array}{l}\sum _{i\in I}\,\sum _{y\in C}\,\sum _{j\in I}\,I{v}_{x,i}^{r}\ast {m}_{i,j,dist(x,y)}^{d}+\sum _{i\in I}\,I{v}_{x,i}^{r}\ast s{l}_{x,i}^{d}+\sum _{i\in I}\,I{v}_{x,i}^{r}\ast o{s}_{x,i}^{d}+E{T}_{x}^{sales}=\frac{Sale{s}_{x}^{r}}{scaled}\,\,\forall x\end{array}$$

The superscript *r* indicates published data. The parameter $I{v}_{x,i}^{r}$ is the swine inventory in the sub-population (*x*, *i*), and $Sale{s}_{x}^{r}$ represents the total sales from county *x* in a year. The parameter *scaled* is used to convert the timescale, this parameter allows us to convert the timescale from yearly to weekly basis. $E{T}_{x}^{sales}$ is the error term for the constraint 3.

The constraint for the slaughtered swine is,

4 $$\sum _{x\in C}\,\sum _{i\in I}\,I{v}_{x,i}^{r}\ast s{l}_{x,i}^{d}+E{T}^{sl}=\frac{TotalSlaughtere{d}^{r}}{scaled}$$

The term *TotalSlaughtered*^*r*^ represents the total number of slaughtered in a year in the system, and *ET*^*sl*^ is the error term for slaughtered data.

The constraint for the sales to the outside of the state is;

5 $$\sum _{x\in C}\,\sum _{i\in I}\,I{v}_{x,i}^{r}\ast o{s}_{x,i}^{d}+E{T}^{out}=\frac{TotalOutshipmen{t}^{r}}{scaled}$$

The term *TotalOutshipment*^*r*^ is the total sales to the outside of the state in a year, and *ET*^*out*^ is the error term for outshipment.

The constraint for the inshipments from the outside of the state is;

6 $$\sum _{x\in C}\,\sum _{i\in I}\,I{v}_{x,i}^{r}\ast i{s}_{x,i}^{d}+E{T}^{in}=\frac{TotalInshipmen{t}^{r}}{scaled}$$

The parameter $i{s}_{x,i}^{d}$ indicates the inshipment probability in a week from outside of the state to the sub-population (*x*, *i*), *TotalInshipment*^*r*^ is the inshipment from outside in a year in the system, and *ET*^*in*^ is the error term for inshipment.

The constraint for the death or lost is,

7 $$\sum _{x\in C}\,\sum _{i\in I}\,I{v}_{x,i}^{r}\ast l{t}_{x,i}^{d}+E{T}^{lt}=\frac{TotalLos{t}^{r}}{scaled}$$

The term *TotalLost*^*r*^ represents the total number of death or lost in a year from the system, and *ET*^*lt*^ is the error term for this constraint.

We assume that the population or inventory size of a sub-population remain constant throughout the year. Therefore, in a sub-population, the summation of the outgoing flows from the sub-population (solid black lines in Fig. 1) is equal to the summation of the incoming flows into the sub-population (dotted red lines in Fig. 1). Constraints for the population conservation are,

8 $$\begin{array}{l}I{v}_{x,i}^{r}\ast [\sum _{y\in C}\sum _{j\in I}{m}_{i,j,dist(x,y)}^{d}]+I{v}_{x,i}^{r}\ast s{l}_{x,i}^{d}+I{v}_{x,i}^{r}\ast l{t}_{x,i}^{d}+I{v}_{x,i}^{r}\ast o{s}_{x,i}^{d}\\ \,=\sum _{y\in C}\sum _{j\in I}I{v}_{y,j}^{r}\ast {m}_{j,i,dist(y,x)}^{d}+I{v}_{x,i,b}^{d}\ast b{t}_{x,i}^{d}+I{v}_{x,i}^{r}\ast i{s}_{x,i}^{d}+E{T}_{x,i}^{pop}\,\,\forall (x,i)\end{array}$$

Here, $I{v}_{x,i,b}^{d}$ represents the breeding population, $b{t}_{x,i}^{d}$ is the probability of birth in the sub-population (*x*, *i*) in a week, and $E{T}_{x,i}^{pop}$ is the error term. The left side of the Eq. 8 is the summation of the outgoing flows from sub-population (*x*, *i*) and the right side is the summation of the incoming flows into the sub-population (*x*, *i*). The range for $b{t}_{x,i}^{d}$ is (7 × 9)/115 − (7 × 12)/112 *week*^−1^, as time period for gestation is 112–115 days and average litter rate is 9–12^25^. The range for $s{l}_{x,i}^{d}$ was chosen based on the lifespan of market pigs in the US, which is about 25 to 28 weeks.

Constraint for the errors is,

9 $$\sum _{x\in C}\,|E{T}_{x}^{sales}|+|E{T}^{sl}|+|E{T}^{in}|+|E{T}^{out}|+|E{T}^{lt}|+\sum _{x\in C}\,\sum _{i\in I}\,|E{T}_{x,i}^{pop}|\,\le {R}_{c}\ast TotalPopulatio{n}^{r}$$

The left side of Eq. 9 represents the summation of all the errors in the optimization problem. Here, *R*_*c*_ is a proportional constant, and *TotalPopulation*^*r*^ is the total swine population in the system. The inequality (Eq. 9) states that the total error in the convex optimization problem should be less than equal to a fraction *R*_*c*_ of the *TotalPopulation*^*r*^. The value of *R*_*c*_ is calculated by using trial and error with an objective to minimize the total error.

Convex cost function (Eq. 1) and constraints (Eqs 2–9) constitute our optimization linear problem. The objective of this estimation problem is to maximize the entropy of the distributions of the objective function of all sub-populations. The performance of entropy measures is sensitive to different factors^31^. Maximum entropy methods can predict accurately given a prior knowledge. However, maximum entropy methods can perform poorly if the prior knowledge is insufficient or inaccurate or contains biases^32^. In our estimation problem, published USDA-NASS data are used as the prior knowledge, and the data was sufficient to solve the formulated convex optimization problem. Maximum entropy methods can also perform poorly if the system changes very rapidly^32^, which is not our case.

### Network development

We develop a network using the movement parameters which are obtained using the maximum entropy optimization. The network development is done in two stages: (1) setup of the population in each farm and (2) setup of the movement links between farms.

In order to generate the network, first, we need the farm level estimates of the pig population. The USDA-NASS data only provide the number of farms in a size range and the number of total pigs in that range in a county. Recorded data on the number of pigs in a farm are generally not available in the US (with the exception of a few counties). To allocate the pig population, we generate random numbers for every farm in a size group *i* within a county *x* with the following constraints:

- The random numbers fall in the range of the corresponding group *i*.
- The sum of all generated numbers is equal to the total number of pigs in that sub-population (*x*, *i*).

The procedure to establish the movement links between farms is inspired by the random network model^33^. Our movement network for pig farms is represented as (*V*, *E*, *W*). The term *V* denotes the set of nodes, the term *E* represents the set of links or connections among individual nodes, and *W* denotes the weight of each link. To generate the movement network among farms, we use the following procedures:

Step 1 For each pig *p*_1_ in a sub-population (*x*, *i*), we generate a random number *rand* from the uniform distribution *U*(0, 1) for sub-population (*y*, *j*), *y* = 1, 2, 3, ….. *C*, and *j* = 1, 2, 3, … *I*. Here, *C* is the number of counties in the system and *I* is the number of size groups.

Step 2 If $rand < ={m}_{i,j,dist(x,y)}^{d}$, a link is created from pig *p*_1_ to another pig *p*_2_. Pig *p*_2_ is picked randomly from the sub-population (*y*, *j*).

Step 3 If there is no link from the parent farm *f*_1_ of pig *p*_1_ to the parent farm *f*_2_ of pig *p*_2_, we create a link *flink* from *f*_1_ to *f*_2_. Otherwise, if a link already exists, we increase its weight by 1.

Step 4 For each sub-population (*x*, *i*), we repeat Steps 1–3.

This process produces a directed weighted network at the farm level. Links or connections among farms represent swine movement. The weight of a link represents the volume of movements occurring from one farm to another.

### Network analysis

To capture the particular features of the developed network, we compute the following network analysis metrics: node strength, betweenness, eigenvector, clustering coefficient, and average shortest path^33–35^. Centrality measures can help us determine the most important or central nodes in a network.

The node strength-centrality measure is the strength of the nodes or sum of the weights of the edges connected to it^36^. In a directed network, the nodes have two types of vertex-strength centralities: (1) in-strength *InS*, and (2) out-strength *OuS*.

10 $$InS(k)=\sum _{l\in NB(k)}\,{w}_{lk}$$

11 $$OuS(k)=\sum _{l\in NB(k)}\,{w}_{kl}$$

Here, *w*_*lk*_ is the connection strength of the edge/link from node *l* to node *k*, *NB*(*k*) is the set of the neighbors of node *k*. Vertex strength can be illuminating in the investigation of diseases spreading. A high in-strength node has a high risk of receiving an infection. On the other hand, a high out-strength node is influential over the network, as such a node can infect many more nodes.

The betweenness centrality measure suggests which nodes are important in the connection flow or act as bridges in the network. Betweenness centrality of a node measures how many shortest paths between different pairs of nodes go through that particular node. The shortest path between two nodes is the path with the fewest number of connections. Nodes with high betweenness centrality have high control over movement flow (here, concerning flow of swine) in the network. Removal of such nodes can effectively reduce connectivity in the network. Knowledge of these nodes can be useful in controlling outbreaks^37^. Let, *p*_*st*_ be the number of shortest paths from *s*∈*N* to *t*∈*N*. We denote, *p*_*st*_(*k*) to be the number of shortest paths from *s* to *t*, that includes node *k* somewhere in between. The betweenness centrality of a node *k* is defined^38^ as:

12 $$B(k)=\sum _{s\ne k\ne t\in N}\,\frac{{p}_{st}(k)}{{p}_{st}}$$

Eigenvector centrality is an extension of the degree/strength centrality. In the eigenvector centrality measure, the centrality of a node is proportional to the sum of the centralities of its neighbors.

13 $$e(k)={\lambda }_{1}^{-1}\ast \sum _{l\in NB(k)}\,e(l)$$

Here, *e*(*k*) is the eigenvector centrality of the node *k*, and *λ*_1_ is the largest eigenvalue of the adjacency matrix [*a*_*kl*_] of the network. Eigenvector centrality of a node can be large if either it has many neighbors or it has important neighbors. Nodes with high eigenvector centralities have high probabilities of becoming infected^39,40^.

The clustering coefficient measures local group cohesiveness. The clustering coefficient *Cc(k)* for a node *k* is the ratio of the number of edges among the neighbors of *k* and the maximum possible number of such edges (for the fully-connected network formed by the neighbors of node *k*). If neighboring nodes of node *k* has *c*_*k*_ connections among them then clustering coefficient can be defined as^35^:

14 $$Cc(k)=\frac{{c}_{k}}{|NB(k)|(|NB(k)|-\mathrm{1)/2}}$$

The average shortest path is the average of the shortest path length between all pairs of nodes in the network.

## Results

### Movement probability estimation

In this research, we solve a convex optimization problem to estimate the swine movement probabilities by using the maximum entropy approach for Iowa. We utilized the AIMMS modeling system^41^ of Paragon Decision Technology to solve this convex optimization problem. The time-scale of our estimation problem is weekly, which we controlled it by using *scaled* = 52 *weeks*/*year*. The boundary of error limit in our system is 5.45% of total swine population in Iowa (*R*_*c*_ = 5.45%). The estimated probabilities are given in Table 1. This table shows swine movement probabilities between size groups for five different distance ranges. The highest movement probability is from size7 to size7 sub-population when the distance between them is less than 20 km. We divide seven size groups into three categories; size: 1–3(small farms), 4–5(medium farms), and 6–7(large farms). From Table 1, we can notice that the movement probabilities from large farms to small farms are small and vice versa.

**Table 1:** Estimated swine movement probabilities *m*_*i*,*j*,*dist*(*x*,*y*)_ × 10^3^ from maximum entropy approach.

Destination								
		Size1	Size2	Size3	Size4	Size5	Size6	Size7
**Distance < 20 ** ***km***								
Source	size1	1.4899	1.3587	1.3890	1.4007	1.4543	1.4641	1.5083
	size2	1.3989	1.5080	1.3755	1.4129	1.4393	1.4611	1.5112
	size3	1.2826	1.1726	1.8054	1.4979	1.5580	1.6066	1.6264
	size4	1.0582	1.1064	1.4199	2.3913	1.7695	1.9519	2.1038
	size5	0	0	0	1.7460	7.1795	6.0844	5.3446
	size6	0	0	0	2.5308	8.7793	14.3449	8.8213
	size7	0	0	0	0	0	0	11.7828
**20 ** ***km*** ** < Distance < 100 ** ***km***								
Source	size1	1.3334	1.3028	1.3834	1.4076	1.4403	1.4511	1.4972
	size2	1.3373	1.2961	1.3767	1.4114	1.4375	1.4463	1.4987
	size3	1.2407	1.1528	1.3516	1.4039	1.5402	1.5589	1.6340
	size4	1.0077	0.7768	1.2906	1.3337	1.7005	1.7403	1.9707
	size5	0	0	0	0.5768	2.4553	3.4121	4.4916
	size6	0	0	0	0	2.0213	4.0961	6.3753
	size7	0	0	0	0	0	0	0
**100 ** ***km*** ** < Distance < 200 ** ***km***								
Source	size1	1.3211	1.2904	1.3840	1.3943	1.4421	1.4449	1.5056
	size2	1.3261	1.3009	1.3899	1.3914	1.4372	1.4392	1.4987
	size3	1.2350	1.1626	1.3534	1.3966	1.4823	1.5003	1.6312
	size4	0.9633	0.7990	1.3194	1.3922	1.6203	1.6701	1.9975
	size5	0	0	0	0.2870	2.0726	2.2576	4.5535
	size6	0	0	0	0	0.7503	1.2075	6.5958
	size7	0	0	0	0	0	0	0
**200 ** ***km*** ** < Distance < 400 ** ***km***								
Source	size1	1.3092	1.2929	1.3708	1.3906	1.4435	1.4587	1.5156
	size2	1.3101	1.2912	1.3705	1.3919	1.4453	1.4608	1.5130
	size3	1.1890	1.1582	1.3361	1.3725	1.4957	1.5190	1.6690
	size4	0.9148	0.8430	1.2363	1.3534	1.6271	1.6868	2.0233
	size5	0	0	0	0.0996	1.9382	2.2667	4.8693
	size6	0	0	0	0	0.1753	0.7087	7.3607
	size7	0	0	0	0	0	0	0
**Distance > 400 ** ***km***								
Source	size1	1.2644	1.2818	1.3040	1.4093	1.4522	1.5169	1.5613
	size2	1.2915	1.2876	1.3032	1.4002	1.4492	1.5108	1.5422
	size3	1.1489	1.1554	1.1864	1.4614	1.4731	1.6829	1.7441
	size4	0.9891	0.8387	0.9770	1.4179	1.6056	1.9855	2.0836
	size5	0	0	0	0.1091	0.8917	3.9986	4.4802
	size6	0	0	0	0	0	3.3953	5.4755
	size7	0	0	0	0	0	0	0.0019

### Network description

We generate a swine movement network for the central agricultural district of Iowa. It has 12 counties: Boone, Dallas, Grundy, Hamilton, Hardin, Jasper, Marshall, Polk, Poweshiek, Story, Tama, and Webster. The total number of farms in those 12 counties is 641, while the net pig population is 2,600,888, which is 12.71% of the total pig population in Iowa. Grundy, Hamilton, Hardin, Jasper, Marshall, and Webster Counties are within the America’s top 100 pork producer counties. Among these, Hardin County is in the 9th position. The descriptions of pig inventories for the above-mentioned counties are provided in the Supplementary Material Dataset 1.

**Figure 2:**
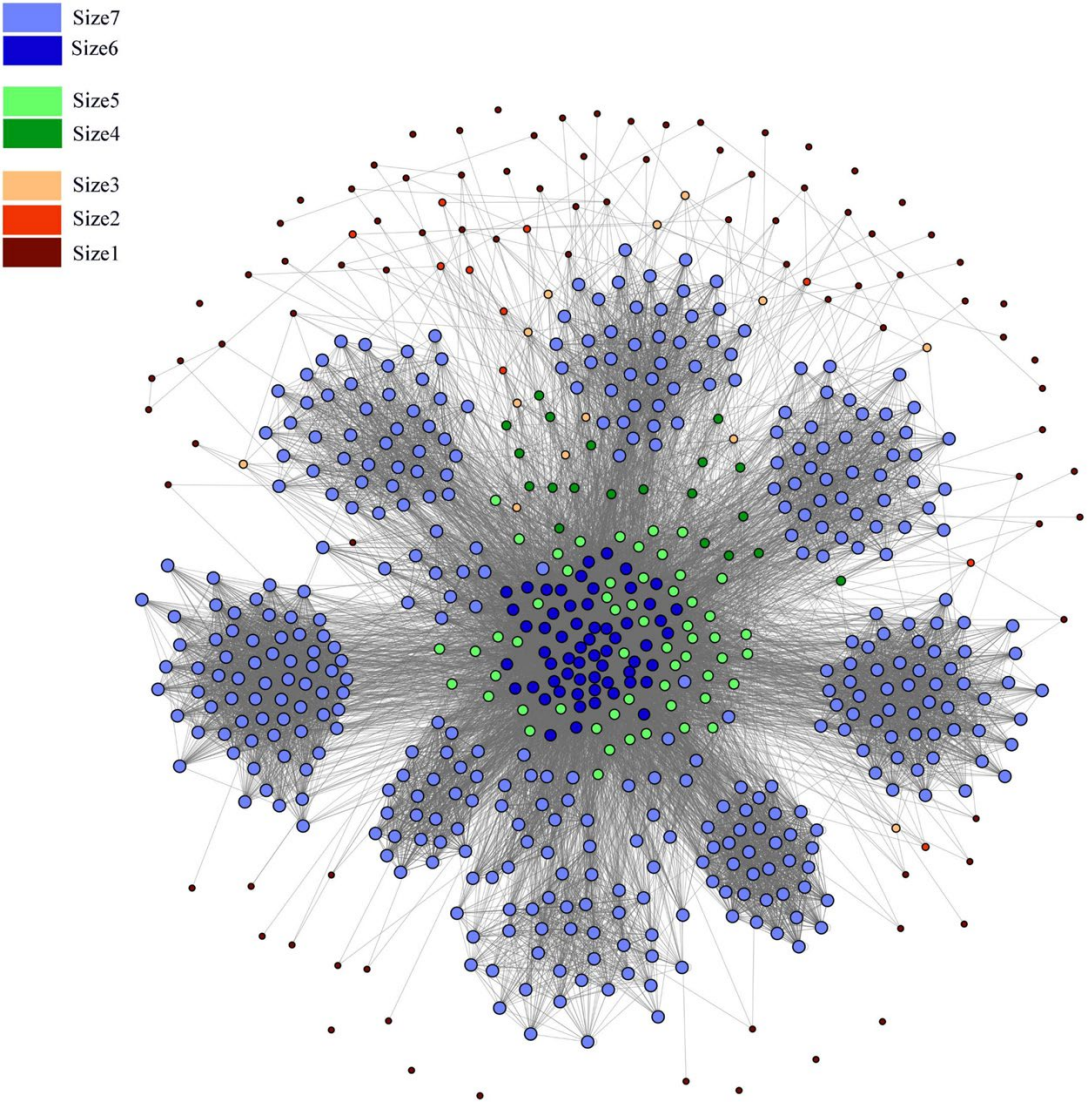
Movement Network for the pig population at the farm level. Different colors represent different size groups. Farms are divided into 7 size groups, size: 1–3(small farms), 4–5(medium farms), and 6–7(large farms).

For these 12 counties, we have developed a movement network (*V*, *E*, *W*), which is shown in Fig. 2. This network is a realization based on the movement probabilities from Table 1. For the network, |*V*| = 641 and |*E*| = 22, 461, the description of the nodes, and the adjacency list for this network is provided in the Supplementary Material Dataset 2 and 3. In Fig. 2, this network has seven types of nodes representing the seven size groups. A description of size groups is presented in Table 2. The largest group is the size7, contains 393 nodes which are presented by light blue. There are 17484 edges among the nodes of this group (67.41% of total edges).

**Table 2:** A summary of the size groups in the network.

Group	No. of nodes	% of the total nodes	No. of edges in a group	% of the total edges
size1	89	13.88%	15	0.07%
size2	10	1.56%	2	0.01%
size3	13	2.03%	9	0.04%
size4	20	3.12%	50	0.22%
size5	56	8.74%	678	3.02%
size6	60	9.36%	1666	7.42%
size7	393	61.31%	12506	55.68%

### Network analysis

The clustering coefficient of the full network is 0.363, the diameter of the network is 7, and the average shortest path length is 2.598. A summary of various centrality measures for the network is provided in Table 3. Node-strength, betweenness, eigenvector and clustering coefficient centrality for seven size groups are presented here. In-strength, out-strength, betweenness, and eigenvector centralities were calculated from the overall network. Clustering coefficients in Table 3 were calculated for networks of the same size group (any node and its neighbors are in the same size group). We used the open source package Gephi to visualize and analyze the network^42^.

**Table 3:** A summary of centrality measures for different size groups in the network.

	Size1	Size2	Size3	Size4	Size5	Size6	Size7
**In-strength**							
mean	1.292	4.700	6.846	16.050	44.304	63.400	151.891
median	1.000	4.000	5.000	15.000	31.500	44.000	100.000
(95% CI)	(0.902, 1.683)	(2.620, 6.780)	(4.214, 9.478)	(11.781, 20.319)	(33.070, 55.537)	(48.566, 78.234)	(135.376, 168.406)
range	(0, 8)	(1, 9)	(1, 17)	(5, 42)	(12, 267)	(18, 347)	(11, 1426)
**Out-strength**							
mean	1.214	4.500	11.385	22.200	55.054	138.450	140.461
median	1.000	3.500	9.000	18.500	53.000	109.5000	90.000
(95% CI)	(0.935, 1.491)	(1.613, 7.386)	(6.746, 16.023)	(16.830, 27.569)	(48.889, 61.217)	(123.497, 153.403)	(122.477, 154.444)
range	(0, 5)	(0, 14)	(2, 26)	(10, 50)	(21, 118)	(66, 282)	(7, 1372)
**Betweenness**							
mean	36.140	386.258	858.157	1531.4	814.294	2390.600	244.137
median	0	86.087	905.169	1289.900	661.0194	2026.000	132.247
(95% CI)	(10.639, 61.642)	(4.551, 767.964)	(413.300, 1303.000)	(1127.400, 1935.300)	(634.840, 993.748)	(1738.500, 3042.600)	(183.130, 305.143)
range	(0, 699.662)	(0, 1237.000)	(14.605, 2388.400)	(228.138, 3189.900)	(48.185, 2715.600)	(324.236, 15229.000)	(0.256, 9932.100)
**Eigenvector**							
mean	0.00086	0.0032	0.0058	0.0326	0.1072	0.1522	0.2381
median	0.00035	0.0030	0.0038	0.0279	0.0854	0.1263	0.1690
(95% CI)	(0.0006, 0.0012)	(0.0020, 0.0044)	(0.0033, 0.0083)	(0.0235, 0.0417)	(0.0899, 0.1245)	(0.1225, 0.1819)	(0.2174, 0.2588)
range	(0, 0.0061)	(0.0011, 0.0064)	(0.00043, 0.0141)	(0.0100, 0.0726)	(0.0281, 0.3391)	(0.0493, 0.6565)	(0.0328, 1)
**Clustering coefficient**							
mean	0	0	0	0.124	0.264	0.449	0.755

**Figure 3:**
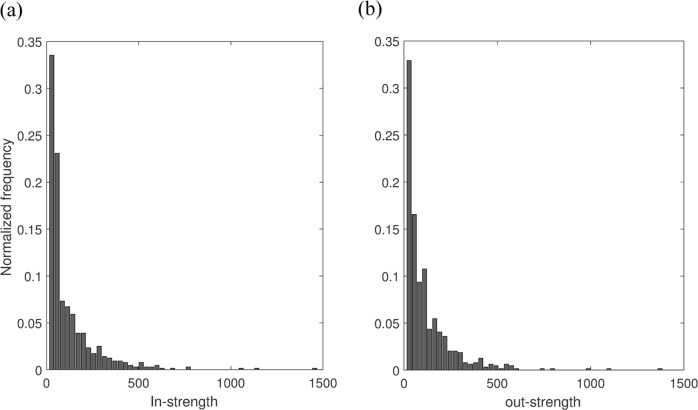
Node strength distribution of the directed network. (**a**) In-strength, (**b**) out-strength.

From the node-strength centrality measures, we observe that the average node-strength is positively correlated with the size groups. Larger size groups have higher average node-strengths. Consequently, size7 has the highest average node-strength (Table 3). The node-strength distribution is provided in Fig. 3. In the network, only a few nodes have high strength and most of the nodes have low strength. This characteristic is similar to the power-law distribution. The range of in-strength is 0–1426. About 90.95% of the total nodes have in-strengths less than 285, which is merely the first 20% of the in-strength range. The range for out-strength is 0–1372. About 91.11% of the total nodes have out-strengths less than 274, which is within the first 20% of the range of out-strength values. The correlation coefficient between in-strength and out-strength is 0.9523, which is an indication of strong correlation.

**Figure 4:**
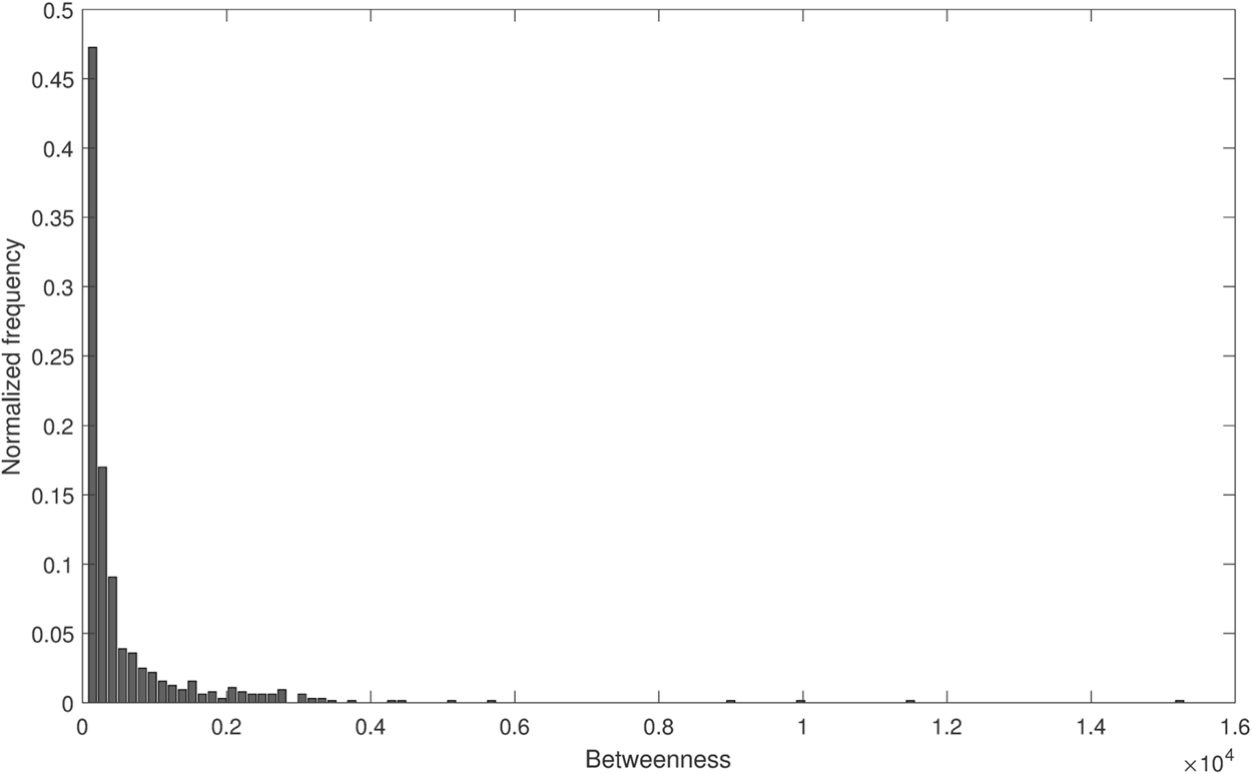
Betweenness distribution of the network.

**Figure 5:**
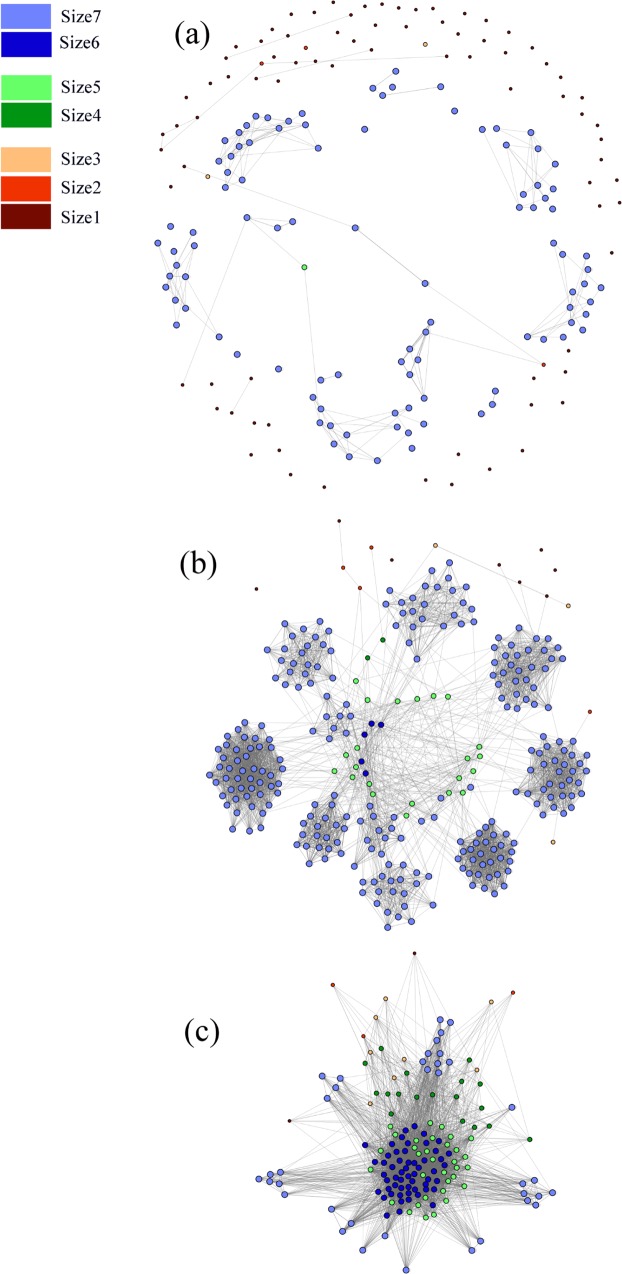
Node groups according to betweenness. (**a**) nodes with low-betweenness, (**b**) nodes with medium-betweenness, and (**c**) nodes with high-betweenness. The connections among visible nodes are presented here.

The betweenness centrality is positively correlated with size groups until group6, after which farms in the group7 have lower betweenness. The farms in group6 have the highest average betweenness. The distribution of betweenness centrality measure is given in Fig. 4. Most of the farms have low betweenness. Few farms act as hubs in the network which have high betweenness. The range for betweenness is 0–15229. We divide the nodes into three groups, (1) low-betweenness (0–50), (2) medium-betweenness (51–500), and (3) high-betweenness (>500). These three groups contain 183, 302, and 156 nodes respectively. These three groups are illustrated in Fig. 5. In the low-betweenness group majority of the nodes are from small size groups, in the medium-betweenness group most of the nodes are from group7, and in the high-betweenness group, most of the nodes are from group6.

**Figure 6:**
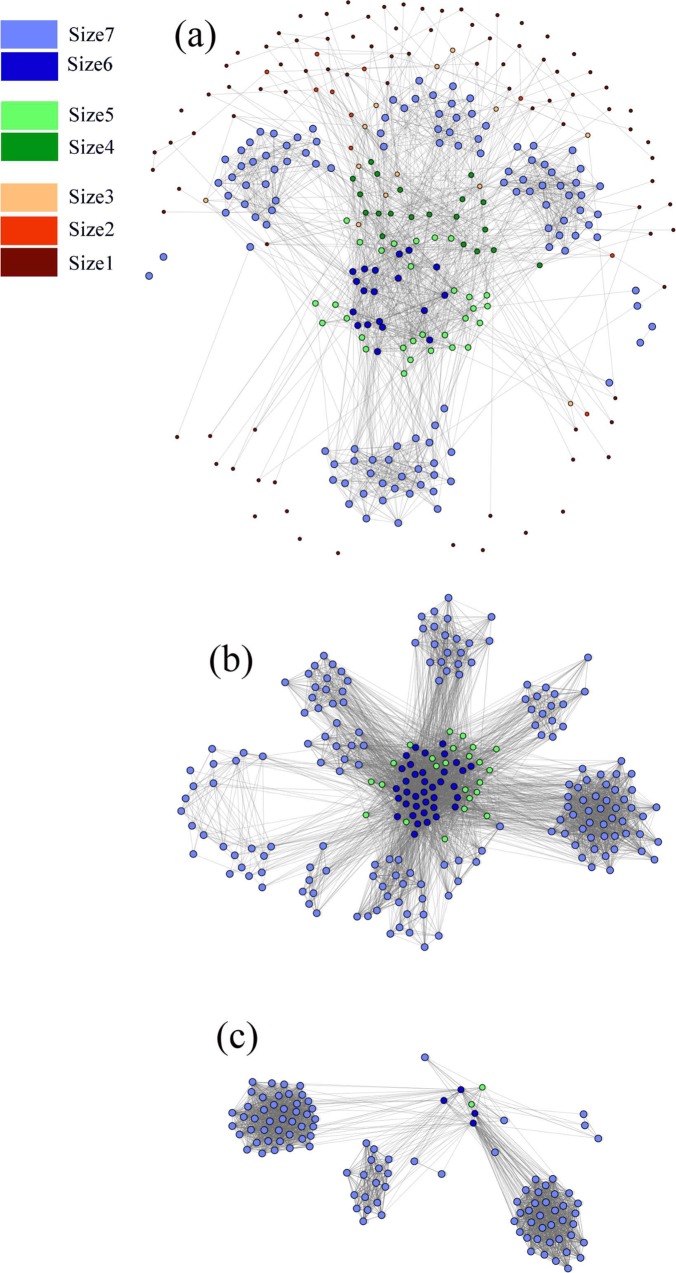
Node groups according to eigenvector centrality, (**a**) low-eigenvector central nodes, (**b**) medium-eigenvector central nodes, and (**c**) high-eigenvector central nodes. The connections among visible nodes are presented here.

The mean eigenvector centrality is positively correlated with the size groups. Larger size groups have higher eigenvector centralities (Table 3). We have divided the nodes (farms) into three groups: (1) low-eigenvector central nodes (0–0.1), (2) medium-eigenvector central nodes (0.11–0.3), and (3) high-eigenvector central nodes (0.31–1). The low-eigenvector central group consists of 298 nodes, the medium group consists of 233 nodes, and the high group contains the rest of the nodes. The network for different eigenvector groups is presented in Fig. 6. Clustering coefficient for group size 7 is 0.755, which is quite high. The nodes from this group form several clusters, which are quite visible in Figs 2 and 6.

**Figure 7:**
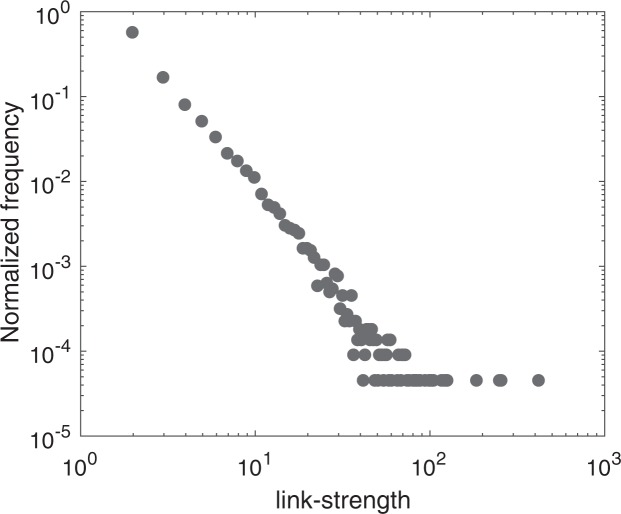
Link-strength or connection-weight distribution of the network. Log-log scale has used for better visualization.

In the network, the importance of links is another useful topic to study^17^. From the link strength or weight distribution, we can see that the majority of the links have a low weight however very few links have high weight (Fig. 7). A link with high-weight represents a high volume swine movement. For a susceptible farm, an infected neighbor connected by a high-strength-link is riskier than an infected neighbor connected by a low-strength-link.

## Discussion

In this study, we have three objectives: (1) we compute optimal estimates swine movement probabilities among counties from the aggregated data of USDA-NASS, (2) we develop a realization of the network from the estimated probabilities, and (3) we analyze the developed network with different network analysis metrics.

Animal movement has been one of the major causes of diseases spread among farms for several outbreaks in the US swine industry. A better understanding of the swine movement network can increase the feasibility of planning effective mitigation strategies that can reduce the risk of disease spread. There is no mandatory animal movement tracking system in the US due to the industry preference for privacy in the swine business. We have estimated the movements among different swine sub-populations using a convex optimization problem, have formulated according to the USDA-NASS data. The discrepancy from our optimization problem is about 5.45% of the total swine population, which is slightly higher than that of a similar work on cattle movement probability estimation^9^ due to a greater amount of data available for cattle. Our estimation can be improved if more data are available. The additional data that would improve the results most is the type of swine operations (for example, nursery, farrow-to-feeder, farrow-to-wean, farrow-to-finish, finish only etc.) at the county level. The USDA-NASS department can collect and publish this information in future reports, as this additional data would not hamper the anonymity of the Census of Agriculture yet greatly improve movement estimations.

The network development algorithm can provide us a realization of the network from the estimated movement probabilities. The generated swine movement network was well connected with a giant component containing 95.94% of the farms. The implication of this high connectivity is that the swine industry may be vulnerable to infectious diseases. All the disconnected farms were smaller farms (inventory size less than 100) where most of them produce meat for their own consumption (60.5% of all small swine farms)^43^. In addition to that, most of these small farms are engaged in all of the phases of swine production (farrow-to-finish producers)^44^. On the other hand, larger farms have more connections among them. One possible reason could be that most of the large farms are specialized in a single production phase to increase productivity^45,46^. Consequently, pig shipments are very frequent among them.

We use centrality measures to understand the characteristics of the movement network. From the analysis of the node-strength centrality measure, we notice that many nodes in the network have low node-strength however very few nodes have high node-strength, who work as hubs in the network. The node-strength distribution of the network is similar to that of scale-free networks (Fig. 3). Compared to a random network, epidemics can spread faster in a scale-free network. In addition to that, scale-free networks have lower epidemic threshold than comparable random networks^47^. This information could be useful because targeted vaccination/node-removal is more effective in scale-free structures than random vaccination^48^. The vaccination, or culling, or quarantine of the hubs (farms with high node-strength) can be crucial to control an epidemic.

If we analyze the average shortest path length and the clustering coefficient of the overall network, we see evidence of the small-world phenomenon in the network. The average path length was similar and clustering coefficient was more than six times larger compared to the similar properties of the equivalent Erdos-Renyi random network^49^, which satisfy the sufficient conditions for small-world properties of the network^50^. The US swine movement network structure is quite vulnerable to any pathogen spreading because of its small-world nature. This result is similar to other studies as well^20–22^. This network has high local clustering. Size7 group (larger operations: headcount is more than 1000) has the highest amount of local clustering (Figs 2 and 6). Therefore, large operations are highly interconnected, making them more vulnerable to outbreaks. Moreover, the structure of the US swine industry has been changing over several years. The number of large operations is increasing, where most of them specialize in one particular phase of production. These changes are increasing the risk for disease outbreaks in the swine industry.

The correlation between in-strength (incoming movements) and out-strength (outgoing movements) is strong. The nodes with high out-strength values also have high in-strength values. This is an important indicator as the nodes with a high risk of receiving infection are also highly capable of spreading them.

Although the group size7 (largest operations) has the highest values of node-strength, clustering coefficient, and eigenvector centralities it is not necessarily highest in terms of the betweenness centrality measure. We found that group size6 has the highest betweenness centrality values (Table 3). The groups size4 and size5 also show high betweenness. The above-mentioned properties indicate that the group size7 forms various clusters in the network, where the operations are highly connected. The operations of medium size, however, maintain the connectivity among the clusters of the largest group. Hence, these medium size operations play a key role in the system. During an epidemic, it is possible to use these high betweenness farms to disconnect the movement network and confine the disease in a smaller part of the network.

We make several assumptions to simplify our model as all necessary data are not available. We assume that the inventory size of the operations is constant on a year-to-year basis. We also consider that movement flows are the same throughout the year because of the resolution limitation of the available data. However, movement flows can be different from one season to another season. The movement flows also can be sensitive to other factors, for example, production technology, business strategy, and food availability. However, we do not have specific knowledge about these factors at this point and inclusion of too many unknown factors increases the complexity of the estimation problem given the limited data. Our estimation steps can be easily adapted by adding more constraints when more data are available.

One immediate use of this network could be the investigation of the stochastic spreading processes^51–55^. This kind of study can help us understand the underlying mechanisms and threshold conditions of spreading processes for various swine diseases including porcine reproductive and respiratory syndrome (PRRS), classical swine fever (CSF), African swine fever (ASF) and many more.

In summary, we present a maximum entropy approach to estimate the swine movement network from aggregated anonymous census data. This method can be used to estimate movement probabilities of other farm animals too for various locations.

## Supplementary information

Dataset 1

Dataset 2

Dataset 3

## Data Availability

The dataset used to perform this research is available from https://quickstats.nass.usda.gov/, https://quickstats.nass.usda.gov/. The authors are willing to provide further details upon request.
